# How did organ donation in Israel become a club membership model? From civic to communal solidarity in organ sharing

**DOI:** 10.1007/s40592-023-00179-7

**Published:** 2023-09-09

**Authors:** Hagai Boas

**Affiliations:** 1 Ben Gurion University of the Negev, Beersheba, Israel; 2 The Van Leer Jerusalem Institute, Jerusalem, Israel

**Keywords:** Solidarity, Organ donation, Transplant policy, Altruism, Israel

## Abstract

Figuring out what pushes individuals to become organ donors has become the holy grail of social scientists interested in transplantations. In this paper I concentrate on solidarity as a determinant of organ donation and examine it through the history of organ donation in Israel. By following the history of transplantation policies since 1968 and examining them in relation to different types of solidarities, this paper leads to a nuanced understanding of the ties between solidarity and health policy. Attempts to foster an all-encompassing consensus on the definition of brain death yielded the Transplantation and the Brain-Respiratory Death Laws of 2008. It was hoped that a wide “civic solidarity” would render Israel self-sufficient in its organ economy. However, the failure of the law led to the breakdown of civic solidarity in organ donation. As a result, initiatives such as the priority policy and non-directed living organ donations, developed out of a narrower conception of solidarity. Juxtaposing these initiatives sheds light on macro level processes for policy makers and suggests solidarity as a key bioethical concept to understand organ donation policies.

## Background

Organ transplantation is a socio-cultural event, no less than a medical procedure. The renowned medical technology remains useless without the cooperation of individuals who are willing to become donors. In fact, this highly advanced medical procedure and its sophisticated post-operative treatment, with its cocktail of immunosuppressant drugs, stand in sharp contrast to the stubborn efforts to encourage enough people to sign on organ donor cards, or even to become living organ donors. The yawning gap between the limited (yet slowly expanding) pool of donors and the demand for organ transplantations for a growing number of medical conditions, clearly presents the irony of scientific and medical success, in that its implementation is, in the end, dependent upon the arbitrary good will of good Samaritans.

Figuring out what pushes individuals to become organ donors has therefore become the holy grail of social scientists interested in transplantations. With patients dying on transplantation waiting lists, finding a way to close the gap on organ donation has become the hardest challenge of transplantation medicine. In this paper I concentrate on solidarity as a determinant of organ donation and examine it in the history of organ donation in Israel. Following Prainsack and Buyx (2017), I argue that solidarity is a more adequate concept than altruism to address organ donation (see also Prainsack and Buyx 2017; Siegal and Bonnie 2006). Adopting solidarity rather than altruism as the key bioethical concept in organ donations sends researchers, bioethicists and policy makers to consider that although organ donation is an individual act, its social underpinnings are rooted in feeling of belongingness to a certain group and in a deep sense of community.

However, the term solidarity is a vague concept. It carries descriptive, normative, and analytic meanings that are can be contradictory and ambivalent, different in their analytic units and referred populations (Bayertz 1999, Brunkhorst 2005, Scholz 2008, Dawson and Verweij 2012). In this paper I suggest a nuanced account of how different forms of solidarities interact in the field of organ donations. Specifically, I present a case where one form of solidarity—civic solidarity—is giving way to a more restricted and bounded form of solidarity. In what follows I propose that Israeli policies of organ donation can be classified according to their assumptions on solidarity: from a consensus-based approach of civic solidarity to what I call here a club membership model, which is a form of a more bounded solidarity.

In the following pages, I present the theoretical framework and approach on solidarity in healthcare generally and in organ donations particularly. I then move to analyze the history of transplantation policy and donation trends in Israel, and then conclude by suggesting the need for policy makers to be sensitive to the ethical difficulties that communal based solidarity raise in organ donations, and to adapt a solidarity model that is based on inclusion and is aimed at equity and equality. In this paper, I hope to achieve three goals: to introduce solidarity as a more adequate concept in understanding dynamics in organ donations; by using the Israeli case of organ donations, to present the interrelations between different forms of solidarities and their ethics; and to argue for the benefits of civic solidarity over communal-based solidarity.

## On solidarities, publics, and organ donation

It is only in the last decade that the concept of solidarity has gained momentum in healthcare and public health. A simple count of publications in “pubmed” revealed a surge in papers with the word “solidarity” in their title: from only 94 in 2011 to 560 in 2022.^1^ Nevertheless, solidarity as an analytic concept is unclear and scholars typify the terms according to its different uses (Bayertz 1999, Brunkhorst 2005), historical development (Stjerno 2008, Ewald 2020) and political meaning (Banting and Kymlicka 2017, Scholz 2008). In healthcare and public health, solidarity is—according to Dawson and Verweij (2012)—“a moral concept in need of clarification”. In their influential work on solidarity in biomedicine, Prainsack and Buyx) define solidarity as “an enacted commitment to carry 'costs' to assist others with whom a person or persons recognize similarity in a relevant aspect” (2017, p. 52). This is a rather general definition that requires clarifications as to the 'cost' that people carry in their acts of solidarity and to the circles of similarity that people feel belong to. “Costs” are forms of contributions that range from time, efforts, money etc. and in the field of healthcare these costs are often coupled with taking a risk such as participating in a medical experiment or in the case of transplant medicine, donating an organ. “Circles of similarity” raises questions as to the scope of these circles, their positioning in a matrix of power relations to other groups, and their coercive force on its members. It seems more constructive, therefore, to speak of solidarities that are set on different registers, which refer to different publics. Scholars differentiate between different modes of solidarity according to their scope: interpersonal, communal, civic, and universal solidarity—all based on the fact that feelings of belonging to a certain group are not uniform and that different publics express different forms of affinity: from parochialism to universal humanism (Heyd 2015). Solidarity, as Banting and Kymlicka (2017) argue is a product of political action and thus reflects certain power relations.

Each form of solidarity is a bounded solidarity. It prescribes boundaries for membership. These can be those of the nation-state, but also of a religion, or any other sort of community. These boundaries can be rigid or elastic, closed or open. Since drawing social lines leaves some out, the question of inclusion/exclusion is always open in discussing solidarity. For the sake of the case presented here, I wish to focus on two forms of solidarity: a communal-based solidarity and a state-level solidarity. The former is understood by Scholz (2008) as “social solidarity”, and the latter as “civic solidarity”. Social solidarity is defined as “a measure of the interdependence among individuals within a group (…) social solidarity pertains to group cohesiveness. But this simple statement belies a much more complicated structure that entails some degree of shared consciousness, shared experience, or some other uniting feature among group members. Social solidarity is a community relation that also entails some binding obligations”. (Scholz 2008, p. 21). Civic solidarity, according to Scholtz, is “the obligations the state as a collective has to each citizen; that is, by virtue of their membership in a political state, each citizen is obliged to all other citizens, and vice versa” (ibid, 27).

Civic solidarity is a form of institutionalized solidarity. Healthcare tax is a good example of civic solidarity where mutualism is expressed by the young, the healthy and the better off (temporarily) vouching for the old, the sick and the vulnerable. Civic solidarity comes often in forms of regulations, laws and policy and is the value that underlies welfare policies. Communal solidarity, on the other hand, is more restricted in scope. It is often expressed in informal giving, it is grounded in networking and its members often regard themselves as extended family. Eligibility and entitlement are different in these two forms of solidarity; civic solidarity is circled around the citizen of the nation state while communal based solidarity is narrower and is not confined to civic concepts of equity and equality, and it is perceived here as like a “club membership”.

I find this distinction useful when discussing organ donation. First, organs are sourced from both the living and the dead. Deceased organ donations are the product of individuals willing to take part, unconditionally and non-directly, in the national effort to find organs for needed patients. This form of conduct results from a sense of belonging to the general group of citizens and can be understood as “civic solidarity”. Living organ donations, however, are the product of private efforts of patients and their close social circle to find a suitable donor. Such a donor is often one of the patient's relatives, but—as shall be seen later—can also be from the patient's community. Such donors act based on “social solidarity”, a communal-based solidarity that unlike “civic solidarity” is based on in-group sentiments of familiarity.

Examining organ donation trends as resulting from different modes of solidarity sets the researcher on a different course. First, the unit of analysis is not the individual but rather the publics (sectors, social groups within a society), their self-interactions and group conflicts. Second, the key term is not altruism, which is an individual-based concept, but rather solidarity. Whereas many studies, from different disciplines, suggest ways of appealing to individuals' altruism in order to encourage organ donation, a solidarity-based approach conceives altruism to be the result, not the cause, of one's social standing, communal ties, and social capital (Guttman et al. 2020). Third, applying a nuanced approach of solidarity in analyzing organ donation trends unearths processes and connections that are hardly seen from the standpoint of individualistic approaches, which are focused on motivations as product of personhood, social psychology, or game theory, as altruism is often studied.

## Methods

Inspired by the works of health historians (Porter 2005; Brandt 2020), this paper introduces a political sociology analysis of organ donation in Israel. The focus is on the interplay between transplantation policies and their socio-political contexts, Specifically, their interactions with different sets of solidarities. This history was divided into three time periods according to changes in transplantation policies: 1968–2008; the 2008 laws; and 2008–present day. The analysis follows a macro-level, historical method of unfolding the events in relation to the examined category.

### A social history of organ donation in Israel

#### From the starting point of 1968 to the double legislation of 2008

The history of organ transplantations in Israel dates to the late 1960s when Dr. Morris Levy carried out a heart transplant in 1968 at one of the Israel’s major hospitals, only a year after the world’s first ever heart transplant in South Africa. A local scandal erupted when it was revealed that the transplanted heart was taken from the deceased without any recorded consent, and evidently without consent from the family either. This incident can be counted as the starting point of transplantation ethics in Israel; the first scandal that would map future transplantation ethics. Two main features of transplantation ethics in Israel were introduced already then: (a) the open linkage between organ donation and brain death, and (b) The religious-secular conflict in Israeli society became the main socio-political arena for debating and regulating the ethics of organ transplantation in Israel.

Nevertheless, it was openly stated, all along the way, that the problem of reaching an agreement on the definition of brain death is connected to the problem of boosting the organ donation rate (Boas and Lavi 2018). This linkage—between the definition of brain death and organ donation—was most clearly illustrated when the legislation of “The Transplantation Law” and “The Respiratory-Brain Death Law” passed on the same day in March 2008.^2^ The juxtaposition of the two laws could be seen as “a package deal” in which a consensus on the definition of brain death would yield a wide consensus that would base a self-sufficient organ economy. Reaching a compromise on the definition of brain death would persuade opponents of the conventional brain death definition to sign on donor cards and to donate organs. The premise was that by unlocking the opposition to brain death, Israeli organ donation rates would rise dramatically.

Up until 2008, Organ procurement was based on a series of laws and regulations that reflected an approach which takes for granted the participation of all members of society in advancing medical science and therapeutic objectives. This approach can be pertained to the abovementioned concept of “civic solidarity.” Procuring organs from individuals, and then allocating them as “public goods” to the first patient in need, reflects the social bond of citizenship that grants mutual protection to all. Signing donor cards indicates that one is willing, unconditionally, to participate in the collective effort to save lives of anonymous end-stage patients. Since they became routine medicine in the late 1980s, transplant surgeries and post-operative care were included in Israeli national insurance schemes, and this rendered organ transplantations economically accessible to all Israeli citizens. This is another index of the notion of civic solidarity as a key ethical value in transplantation policies in Israel.

But reaching a satisfactory level of civic solidarity that would yield enough organs for transplantations was never achieved. Side by side with the struggle over the definition of brain death, the shortage of organs exacerbated. Israeli patients, mainly renal patients, turned during the 1990s to find organs through trafficking (Budiani-Saberi and Delmonico 2008; Mor and Boas 2005). During those years, the high times of neoliberal globalization, organ trafficking became one of the main challenges of transplantation medicine.

#### The laws of 2008

The Israeli government and legislator were determined to remove Israel from the blacklist of human law violators and to adopt a regulatory scheme that would be in line with liberal values. The Transplantation Law and the Brain-Respiratory Law were enacted on the same day, March 24, 2008, as one legal package coupling brain death and organ donations in an unprecedented manner. Up until 2008, similar to organ donation policies, brain death was determined by hospital committees and, due to the ongoing controversy about the legitimacy of brain death, enacting a binding law was hindered. The aim of the laws was to simultaneously bring an end to organ trafficking and to compensate the inevitable drop in organ supply by mainstreaming the brain death criterion. The new brain death law was dubbed “The Brain-Respiratory Law,” that determines death according to the absence of brain stem function, responsible for respiration. The 2008 law added some important nuances. The new law adopted the resolution that death could be determined when the total irreversible loss of the brain stem, responsible for respiration, is confirmed.

The regulation of transplantation policy was also debated for years. The Ministry of Health tried to enact the Transplantation Law in 2003, and the Israeli parliament debated the act for years until its enactment together with the Brain-Respiratory Death Law in 2008. Its importance lies in combatting organ trafficking by criminalizing involvement of Israeli citizens (aside from the patients and vendors) in such activity, with a penalty of up to 3 years in prison. Further, the law forbade HMOs, insurance companies, and any other institutions from reimbursing patients for organ transplantations outside Israel (with few exceptions).

Closing the door on organ tourism, however, implied an even worse local organ shortage. The trade-off between conforming to the international ethical standard and exacerbating organ shortage could only be mitigated if the national transplantation center enlarged the pool of deceased donations in what can be termed as a self-sustained organ economy. In fact, the national transplantation center, as a state apparatus, could exercise its procurement techniques only on deceased donations. The center could regulate living donations as well as controlling them, but it could not order someone to become a living organ donor. This left the sole option of deceased donations as the main field of such a self-sustained organ economy. Balancing the trade-offs between combatting organ trafficking and enlarging the potential pool of donors could only be achieved—for the state—by increasing consent for donations. Reaching an agreement regarding the definition of brain death was needed in order to serve as a guarantee against exacerbating the organ shortage. The two laws were to act in tandem to bring Israeli transplantation medicine closer to the organ-supply standards of developed countries.

The legislation of the double laws was based on the sociological conception that characterized the Israeli debate from its onset in the late 1960; that an endorsement from the orthodox rabbis would drive most Israelis—secular and religious alike—to sign organ donor cards. This was the basis of hope for building organ donation economy based on civic solidarity that would encompass all Israelis alike and would benefit patients on the waiting lists. It was hoped that reaching a consensus on the issue of brain death would yield a massive wave of donor cards from all streams of Israeli society. The goal was establishing a basis that would satisfy the majority of the public and would mitigate the controversy over brain death.

This hope, however, failed. Although Orthodox rabbis—mostly those who are fully committed to Israeli statehood—approved the new definition of death and called on their congregations to sign on organ donor cards, other circles of Orthodox rabbis did not issue any approval that would recognize the “kosher” brain death compromise. Although marginal in numbers, the influence of these rabbis is vast and plays on previous universal fears of brain death and organ donation (Boas and Lavi 2018). In such a contested terrain, as in the realm of death definition and body-part removal, the voices of the most conservative prevailed. The concept of a self-sufficient economy, based on wide civic solidarity, in which deceased organ donation would substitute for the forbidden flow of trafficked organs, was proved wrong. Now there was no real answer for patients on the waiting lists, as organ donation rates remained low. Civic solidarity through organ donor cards was at best poor and insufficient for many on the waiting lists.

Despite some preliminary optimistic estimations (Lavee et al. 2013), there was no dramatic increase in the rate of post-mortem organ donation after the legislation of the “package deal.” Comparing the waiting lists for transplantations indicates otherwise: In the year of the legislation, 864 patients were registered to transplantations. This number increased to 1266 in 2020 while the number of deceased transplantations did not increase in a similar way. The number of hearts, for instance, that were transplanted in 2008 remained at the same level even 13 years following “package deal”.^3^ The Brain Death Act, with its detailed protocol, did not convince the leaders of more extreme circles of Orthodox Jews to support organ donation. Although a long list of Orthodox rabbis did openly express support of this law, the influence of the law's opponents depicted the controversy as still unsettled. Without reaching a fully consensual solution to the brain death problem, the connection between brain death and organ donation was again politicized. It reentered the public sphere, ready to be debated again. The history of organ donation trends and policy after 2008 is marked by abandoning efforts to obtain organ donation through appealing to consensus, trust, and the building of civic solidarity. Instead. Narrower and group-oriented forms of social solidarity emerged. Instead of bridging conflicts and resolve discords, each camp fostered its own position on organ donations, hoping to yield more donations to its members.

## Towards a club model of organ donation

### The priority policy

The failure of the two new laws to boost donation rates led to a counter-reaction of unpacking the deal. The priority or the “points plan” seems as if proponents of the brain death criterion decided to take measures against individuals who refused to donate organs. Specifically, a policy of assigning priority points on waiting lists to donor card holders, actual donors and their relatives was introduced and became operative in 2012. The idea itself was already suggested in the early 2000s (Steinberg 2004). In 2004 prominent bioethicist Robert Veatch supported such a proposal for being “fair and equitable to provide some acknowledgment of altruistic actions” (Veatch 2004, p. 2). Although not restricted to deceased donation, this policy line again merged consent with the definition of brain death and the problem of organ procurement. The principle is simple: those who sign an organ donor card, as well as their family members, gain an additional point on the waiting list to receive an organ (Quigley et al. 2012; Lavee et al. 2013). Living donors who donate to family members or acquaintances are also granted extra points on the waiting lists for organ transplantation. Instead of reaching out to those who find it difficult to accept the brain death criterion, policy makers moved to foster an in-group sentiment of solidarity between individuals who share the same values.

The legal background of the measure is section 9(b) of the Organ Transplantation Law, which stipulates that one of the functions of the National Transplantation Center Steering Committee is to advise the health minister on formulating policy. The steering committee led by Prof. Jacob (Jay) Lavee, a cardiologist and heart transplantation specialist at Sheba Medical Center-Tel Hashomer, proposed the new regulation giving priority to donor card holders. Prof. Lavee recounts his immediate motivation for creating the priority regulations:

It all started with... a case that shocked me, and that made me decide that we had to change the approach to organ donation from the ground up. It involved a heart transplantation candidate who was hospitalized in my department for a long time in serious condition and, as a result, was placed at the head of the waiting list. He turned to me one day and confessed in all honesty that if, God forbid, the situation were reversed, and he was asked to give his agreement to donate the organs of his loved one who had died, he would refuse to grant this consent based on his beliefs and the advice of his rabbi. Even though I appreciated his honesty, the basic injustice and immorality of his words infuriated me and would not let me rest. (…) Despite the legitimate criticism, I fought stubbornly for the idea [of priority [for donor card holders], since in my eyes it had the potential to provide a suitable answer to the widespread phenomenon in Israel of 'free riders'—the large number of people who openly declare that they are opposed to organ donation but who do not shy away from accepting organs from others in time of need (Lavee 2013).

I quote Dr. Lavee at length not only because of his central role in setting the process in motion, but also because he clearly expresses the transition from civic to communal based solidarity as the basis for organ donation. Whereas civic solidarity seeks a wide consensus and up until the 2008 package deal, attempts to reach a compromise were sought on the assumption of such solidarity, the prioritization policy is a result of singling out those who do not consent to the definition of brain death.

Furthermore, Lavee’s usage of “free riders” marks the boundaries of communal lsolidarity in terms of shared burden. This idea of punishing free riders was found as a central justification that transplant surgeons and other medical practitioners expressed in supporting this policy (Guttman et al. 2020). In the collective Israeli imagination, the label of “free rider” or “parasite” carries additional implications that once again feed into the religious-secular tension and into the question of “sharing the burden,” to use the current Israeli term. This choice of words can be seen as the point where encompassing solidarity is conceived as unattainable, since not all “arry the burden” of alleviating the organ shortage. In their discussion on solidarity in diverse societies, Banting and Kymlicka (2017) argue that “free-riders” arguments are weakening the legitimacy of state level, open-to-all, social programs and foster narrower, communal-based sentiments of solidarity.

The suggested model, based on the criterion of “burden-sharing,” is universally applied at its formal level: any citizen—secular, religious, ultra-Orthodox, Jew or non-Jew—who does not have a donor card does not receive an additional point. Although its open to all jargon, in practice the assumption of universality is mistaken. The underlying assumption of individuals that calculate their preferences regarding bodily practices as organ donations outside cultural, religious and political contexts is heavily biased in favor of the secular population.

The “if you sign, move up the line” measure reflects the image of an individualistic society, of people who act based on independent will, pure calculation, and with full and comprehensive information available to them. In other words, it is a move in which the liberal-secular ideology of utilitarian individualism presents its hierarchy of values and relates to it as a universal ethical agenda. It is an act of protest in the face of the politics of accommodation, which has failed repeatedly to find a broad consensus in the matter of determining brain death. In fact, when asked lay people about their ethical concerns about the priority policy, some consider it as anti-solidarity and aimed against individuals whose beliefs prevent them from signing on donor cards (Guttman et al. 2011). In a later study, Guttman et al. (2020) reviewed the accumulating ethical concerns vis-à-vis Israeli transplant surgeons and other medical practitioners' views and found the policy ethically and practically problematic. They concluded by indicating that 15 years after its enactment that “no dramatic change has occurred” (ibid, 536).

Furthermore, implementing the prioritization policy required an ethical compromise. According to a long and respected medical tradition, the only criteria for granting medical treatment are medical standards. Doctors do not distinguish between patients based on their altruistic virtues, and treatment should not be based on non-medical considerations (Barilan 2014). In a recent study, Elalouf et al. (2020) found that medical criteria are more important to the Israeli public in setting criteria for allocating organs than whether the candidate is registered as potential organ donor. Berzon (2018) introduces six other ethical challenges to the priority model. She concludes that such a model can be fair only in a soft opt-out model where the default mode is to donate organs and hence to enjoy the priority points. Yet, the gist of the priority points is that they work only if just a section of society abides. If all receive the same bonus points the advantage evaporates. This simple truism attests even more to the restrictive nature of the priority policy, and to its “club membership” underpinning concept.

### The “Gift of Life” (Matnat Chaim) organ matching organization

The prioritization policy was read as a direct confrontation with those parts of the population who choose not to donate post-mortem organs due to their objection to the brain death criterion. However, it was those circles that actually saved the Israeli organ economy from total disintegration by boosting living organ donations. The emergence of the altruistic Orthodox Jewish anonymous living donor in the second decade of the 2000s was an unexpected twist, and yet it played along the known lines of organ transplantation as implicated in the political culture of Israeli Jewish society. These donations, also defined as Non-Directed Living Donations (NDLD), are exceptional form of organ supply. In the US the percentage of such donations increased from only 1% in 2002 to 7% in 2020 out of all living organ donations,^4^ in Israel, with Matnat Chaim the main supplier of NDLD, most of living donation events are of this kind.^5^ In 2022 it was indicated that Israel leads the world in non-directed organ donations.

The late Rabbi Yeshayahu Heber was a kidney recipient who founded Matnat Chaim (“Gift of Life”) in 2009 in memory of a young person who died while he was waiting for a kidney transplant. The number of dialysis patients waiting for transplantations in 2009 was about 800. By his death in March 2020, he had fulfilled his life mission and recruited in about 10 years 800 volunteers for kidney donation to strangers. By the end of 2022, Matnat Chaim procured over 1400 volunteers. This unprecedented phenomenon cannot be explained outside the context of Israeli society and is rooted in the particularities of sociological structure of Orthodox communities as well as in the notion of Jewish mutual support. Sociologically, this achievement is the product of another example of social solidarity.

Similar to parallel organizations of matching patients with donors, this nonprofit private organization runs a list of people willing to donate a kidney to someone they don’t know. The procurement process of volunteers is in informal cooperation with medical center and transplantation units. Patients in need of a kidney transplantation register with the organization, and volunteers to donate organs, who wish to donate to patients they are not acquainted with, are sent by the organization to undergo a series of physical tests at the transplant coordination units in hospitals. The volunteers are recruited by targeted campaigns firstly in Orthodox communities, but lately in expanding circles of Israeli society. Still, the majority of volunteers come from the Orthodox and ultra-Orthodox populations.

From data gathered by the organization (n = 209), only around 5% of volunteers identified as “secular,” while 95% identified themselves either as “national-Jews” (observant Jews) (66%), Haredi (ultra-Orthodox) (27%), and traditional. Only one donor identified as an atheist. This overrepresentation of the Orthodox population corresponds with another dominant feature: 49% of donors live outside the Green Line, i.e. in settlements in the Occupied West Bank (not including Jerusalem). These distinct sociological features were also found in a study of Matnat Chaim (Kurleto et al. 2020). 98% of their sample (n = 180) reported that religious belief was their highest life priority (followed by family, 96%, more than one answer could be chosen).

The distinct religious character of Matnat Chaim derives naturally from the personality and social milieu of Rabbi Heber. Rabbi Heber succeeded in winning the support of those ultra-Orthodox rabbis who consistently opposed any compromise over the brain death debate. With the support of those rabbis, he managed to turn high level of religiosity from an inhibitor to organ donation to one of its main facilitators. The “Gift of Life” campaigns are mostly concentrated in religious communities, with its brochures and publications appearing in the community papers mainly during the weekends and on holidays. Thus in 2012, after the organization enlisted only a few dozens of donors, the Haredi paper “Yated Neeman” printed the following:

People find it hard to believe! Yet, this is a fact! Many dozens of yeshiva students, women, young and old, have donated a kidney in the last two years, voluntarily, for nothing, to people they do not know. After the holiday, the eightieth transplantation from a donation procured by the organization is going to be performed. The donor is an industrious and diligent yeshiva student, and the recipient is the wife of another student, from another town, the mother of a large family, who will receive one of his kidneys, without any prior acquaintance between the two (…). It is possible that your neighbor in the next building or the secretary in the office have humbly, for the sake of heaven, donated a kidney, and this a great deed in our times of repentance (Hevroni 2012).

After Rabbi Heber’s unfortunate demise due to Covid-19 in March 2020, his widow Rachel stepped into the void and took the lead of the organization and was able to procure dozens of volunteers in a short period of time. In Israeli numbers, the success of Matnat Chaim is even more significant. The efforts of the Israeli Transplantation Center to enlist anonymous living donors and post-mortem donations lag far behind. Contrary to the stiff opposition to the brain death definition among ultra-Orthodox circles, that hindered the “package deal” of 2008, living kidney donation is considered a good deed, “*a mitzvah*” (Kurleto et al. 2020).

But the eagerness to donate is not unconditional. On the registration form, under the clause “comments and requests,” the candidates for donation may specify the general demographics of their prospective beneficiary. This can be on a gender basis (religious tend to donate within their own gender, despite the example above), age, position on the waiting list, etc. The most controversial and debated condition considers the recipient’s Jewishness. For most of the donors, the recipient can be secular, even a non-believer, but s/he must be a Jew. The organization’s ethical guidelines explain that: “It is the right (and some say the duty) of everyone to prefer those who are closer to them. We do so in endless contexts; organ donation is no exception. It is very well possible that the personal story would touch the heart of a stranger so that he would become a kidney donor.”^6^ The guidelines also add that: “When a donor applies to Matnat Chaim, and volunteers to donate a kidney, the organization asks them if they have preferences as to the identity of the patient they want to donate to. Some prefer to donate to young patients; some prefer to donate to a man or to a woman, some prefer to donate to a Jewish patient, and some prefer to donate to an Arab or Palestinian patient, some would rather donate to patients who would keep a strict diet and have better chances to live longer with the donated kidney. We have encountered endless preferences according to the donor’s subjective choices. Our premise is that the donor's independent choice is totally legitimate, and it is similar to alms, which are also given according to subjective preference. The donor's donation is the gift of life and like any other gift it is their right to choose to whom they give their gift.”^7^

In a personal interview I asked Rabbi Heber (April 2019) about the reasoning of asking the applicants for their preferences. He replied that the organization added the preferences clause in the application form to prevent cases in which the donors, after undergoing all the efforts and pains associated with becoming organ donors, suddenly realized that their kidney recipient is far from what they imagined and hoped for. Rabbi Heber thought that the conditioning aspect is legitimate. Its objective—according to him—is to accommodate the way of life of the recipient to the donor. Eventually—he added—the actual donation, with its particularities, subtracts another patient from the waiting list and by that helps everyone else. A win–win situation. Rabbi Heber also wanted to emphasize that his initiative succeeded in the Jewish religious sector because that is his milieu and he really hoped that parallel projects would be initiated in the secular and Arab sectors.

Yet, despite the wide range of preferences, the religio-national preference, i.e. to donate only to a Jewish patient, emerge as a dominant factor in the actual donations. Rabbi Heber explains this tendency: “Moshe is not willing to donate to someone that might stone him the next day. I find this legitimate. It is his right to prefer his patient according to his own will (…) Some say to me I want to donate only to an observant Jew, and some say to me I want to donate only to a Jewish patient without any further condition” (Rat 2014).

In a report on the organization's convention, the reporter asks one of the donors: “If the goal is to save lives, why the conditioning?” The answer is: “I firstly have concerns regarding my own people. Charity begins at home. If I am already doing this big step and giving something out of myself, I want to give it to someone from my own people. Its natural and it is also my right” (Stern 2016).

For the donors of Matnat Chaim, their prospective beneficiaries are not total strangers. They belong to the general Jewish community. In fact, the closest ethical model in organ transplantation to these acts of donation is not the anonymous altruistic donor, but rather intra-familial donations where the donation is conditioned to someone within one's family. The Matnat Chaim donors simply extend the notion of family to the entire Jewish community. When Matnat Chaim's donors declare: “Charity begins at home,” they emphasize home in communal belonging terms. They would prefer to donate to someone they feel affinity to.

Matnat Chaim is a clear case of solidarity at the communal level, Unlike the priority model that hides its biases, Matnat Chaim clearly draws the boundaries of its solidarity scope. In this sense, the condition clause further enhances the communal sentiment that produces, in turn, more candidate to non-directed donations. Obviously, non-Jews are not likely to benefit from the organization, and not too many non-Jews are even registered as candidates with the organization. Critics of Matnat Chaim accused it as being “racist” in that sense (Epstein 2017). Hilhorst et al. (2005) warn against biases of discrimination and social injustice when implementing such donations. They note that: “in societies where both race and religion have created deep conflicts, the fear of discrimination can be real indeed” and yet they add that “not all preferences regarding donation are based on dubious beliefs that exclude and humiliate. They can reflect a sincere and altruistic wish to help particular others” (Hilhorst et al. 2005, p. 1472). In a sense, this mode of donation is possible only with an organization that can is built upon social solidarity conceptions of social boundaries. Public organizations of organ procurement that are committed to equity, fairness, and equality, and are built upon conceptions of civic solidarity cannot offer potential donors such an exclusionary option.

## Discussion

Israel was the first to adapt the priority model worldwide and Israel leads the world record in non-directed living organ donations, thanks to Matnat Chaim. The priority principle is a mechanism to encourage people to sign donor cards and to boost post-mortem organ donation, while Matnat Chaim acts in the field of living organ donation. Despite this difference in the two, there is much similarity between them. They are both built on a sense of communal solidarity. They both can be seen as clubs rather than open to all options that is based on a wider conception of the public. e. In the priority model, solidarity is more akin to what Dawson and Verweij (2012) understood as “rational solidarity” where one acts towards the other out of a calculus that takes into consideration their own good as well. Matnat Chaim's model of solidarity is closer to their conception of “constitutive solidarity,” where donation results from a deep commitment to one's community. They both, nonetheless, are a break with the conception of civic solidarity that blindly redistributes health resources—organs for transplantations in this case—to the public.

Both the priority model and the Matnat Chaim organization exhibit exclusionary features. Both have a bounded sense of their respective communities: the priority model is designed to give advantage to those who accept the definition of brain death and can therefore participate in the organ donors pool, and Matnat Chaim appeals to the communal sentiment of “all Israel are responsible for each other”. As in any form of solidarity, the communal boundaries are exclusionary: the priority principle can be seen as directed against the ultra-Orthodox population who cannot sign donor cards because they oppose the definition of brain death. In contrast, the Matnat Chaim organization operates based on the exclusionary political culture of Israel that emphasizes Jewish belonging as a defining determinant of its national identity.

Juxtaposing the cases of Matnat Chaim and the priority model indicates the importance of sociological understanding to ethical deliberations in organ donation policies. The analysis supports the classic Durkheimian claim on pre-contractual solidarity (Follert 2020): it is when the sense of solidarity is tangible, when belonging is well defined—as “sharing the burden” of signing organ donor cards in the case of the priority models, or with “charity begins in home” in the Matnat Chaim organization—that individuals are more willing to become organ donors. Furthermore, the Israeli case demonstrates that in a deeply conflicted society, featuring deep diversity between groups on religious, ethnic, and national grounds, the attempt to foster civic solidarity that in turn will yield enough organ donations is difficult. This difficulty worsens when alternative, more communal-based forms of solidarity enter the scene of organ procurement.

## Conclusions

A sociological analysis on organ donation trends suggests a more nuanced perspective to policy makers. It suggests a breakdown of the concept of “the public” to “publics” in the operation of solidarity. Thus, the introduction of such models—as the Israeli case demonstrates—can be good news for hegemonic groups—Jews in the case of Matnat Chaim and full supporters of medical science in the case of the priority model, but bad news for groups outside those lines. When civic solidarity is replaced with communal-based solidarities, the grip of state regulations on equity and equality is weakened; redistribution is then according to local communal norms and the protection of the state on vulnerable groups and their access to social goods is undermined. Furthermore, although ethicists have argued that softening the standards for donations can ease organ scarcity (Saunders 2012), the Israeli case shows empirically what such models entail: the costs for non-hegemonic groups in models that are based on narrow and communal based solidarities rather the inclusive civic solidarity, are too high. Policy makers then should push forward in fostering a civic solidarity approach to organ donation.

Organ donation policies are often caught between the urgency of severe organ shortage and ethical concerns as to where to draw the line that will preserve organ procurement and allocation in a manner conforming with social justice, indiscrimination, and equity. The discussed trends seem to challenge these ethical boundaries. Matnat Chaim is a private matching organization that rests on the fact that it is impossible to dictate to a person who wishes to donate a kidney while alive, to whom this kidney will go. Measures of equity and social justice are not the considerations of private philanthropic agencies but are the fundamental ethics of formal state agencies. The priority model, with its restrictive and exclusionary consequences, is nonetheless a state apparatus. It is built on a narrow understanding of solidarity rather than civic solidarity as expected from a state-led apparatus. Unlike the work of Matnat Chaim which remarkedly improved organ donation rates, the effectiveness of the priority model is questionable both ethically and practically. Given that there is no “one public” in diverse societies such as the Israeli one, the interplay between different sorts of solidarities and policy should be with discretion. Operating in the public sphere, deceased organ donations should be allocated under the premise of civic solidarity; organs should be considered as medical resources provided to the most needed without any “social worth” considerations. Living organ donations, however, are always the product of private endeavors of patients and their families to find a donor from their close social circles. Organizations like Matnat Chaim operate in accordance with this sense of community as an extended family solidarity. It can be hoped that parallel organizations could be established in other sectors to buttress the state-level endeavors to procure organs and the close the gap on organ shortage.

## Footnotes

1 https://pubmed.ncbi.nlm.nih.gov/?term=solidarity (Accessed June. 8th 2023).

2 https://www.nevo.co.il/law_html/law00/4025.htm (Transplantation Act); https://www.nevo.co.il/law_html/law00/4026.htm (The respiratory-Brain Death Law) in Hebrew (Accessed June 8th 2023).

3 https://www.adi.gov.il/ (data in Hebrew) (Accessed June 9th 2023).

4 https://optn.transplant.hrsa.gov/data/view-data-reports/build-advanced/ (Accessed May 10 2020).

5 182 NDD were procured by Matnat Chayim organization. out of the total 273 living donations in Israel. Data on Matnat Chayim The data on NDD was provided in personal communication with the organization.

6 https://kilya.org.il/wp-content/uploads/2016/01/KlaleiEtikaHeb.pdf (in Hebrew) (Accessed June 8th 2023).

7 ibid.

## Declarations

### Availability of data and materials

The interview with R. Heber and data on “Matnat Chaim” are available from the corresponding author upon reasonable request.
